# Retinal Neuropathy in IGT Stage of OLETF Rats: Another Characteristic Change of Diabetic Retinopathy

**DOI:** 10.1155/2021/3181347

**Published:** 2021-10-19

**Authors:** Zhenhong Guo, Xiaoyue Sun, Juhong Yang, Jinlan Xie, Feifei Zhong, Xinran Li, Yi Zhang, Fei Han, Xiaoyun Yang, Shaohua Yang, Wei Zhou, Baocheng Chang

**Affiliations:** ^1^NHC Key Laboratory of Hormones and Development, Tianjin Key Laboratory of Metabolic Diseases, Chu Hsien-I Memorial Hospital & Tianjin Institute of Endocrinology, Tianjin Medical University, Tianjin 300134, China; ^2^Department of Endocrinology and Metabolism, Tianjin Medical University General Hospital, Tianjin 300000, China; ^3^Department of Ophthalmology, Tianjin Medical University General Hospital, Tianjin 300000, China

## Abstract

**Aims:**

We investigated the changes of retinal structure in normal glucose tolerance (NGT), impaired glucose tolerance (IGT), diabetes mellitus (DM), and diabetic kidney disease (DKD) stages in Otsuka Long-Evans Tokushima Fatty (OLETF) rats.

**Methods:**

We assigned OLETF rats to four groups based on their OGTT results and 24 h urinary microalbumin (24 h UMA) levels: NGT, IGT, DM, and DKD groups. We observed the structural and the corresponding pathological changes and quantified the expression of HIF-1*α*, iNOS, NF-*κ*B, VEGF, ICAM-1, and occludin in the retina.

**Results:**

Significant damage to the retinal structure, especially in retinal ganglion cells (RGCs), was observed in the IGT stage. The expression of HIF-1*α*, iNOS, NF-*κ*B, VEGF, and ICAM-1 was significantly upregulated, while that of occludin was downregulated.

**Conclusion:**

Significant retinal neuropathy occurs in the IGT stage. Inflammation and hypoxia may damage the blood retina barrier (BRB), leading to diabetic retinopathy.

## 1. Introduction

With the increasing prevalence of diabetes, chronic complications, especially diabetic kidney disease (DKD) and retinopathy (DR), pose serious social problems threatening human health. The overall prevalence of DR, the primary retinal vascular complication of diabetes mellitus (DM), is 34.6% for any DR in DM. It is a leading cause of vision impairment and blindness in the working-age population [1–3]. DKD is the major cause of end-stage renal disease (ESRD) worldwide. DR is usually accompanied by DKD, and both have similar pathogenesis [4, 5]. Multiple studies have demonstrated a significant correlation between DR and DKD in patients with type 2 diabetes [6–8]. Some studies also believe that DR may precede DKD [9, 10]. Patients may suffer from various degrees of kidney injury before or upon the diagnosis of diabetes [11, 12]. Our previous studies focused on the structural and functional damage to the renal tubule in the impaired glucose tolerance (IGT) stage [13]. As DR may occur at an earlier stage of diabetes, we analyzed the structural alterations of the retina in different stages of diabetes and investigated the pathogenesis of DR in Otsuka Long-Evans Tokushima Fatty (OLETF) rats.

## 2. Materials and Methods

### 2.1. Animals and Groups

The study was performed adhering to the National Institutes of Health (NIH) guidelines for the care and use of laboratory animals (NIH Publication No. 85-23 Rev. 1996) and was approved by the Institutional Animal Care and Use Committee of the Tianjin Medical University.

Thirty-two male OLETF rats were supplied by Otsuka Pharmaceutical Co., Ltd. (Tokushima, Japan). They were housed in an air-conditioned room at 20-25°C with 50%-70% humidity and a 12 h light-dark cycle and fed a high-fat diet. We allowed the rats free access to food and water. Thirty-two male Long-Evans Tokushima Otsuka (LETO) rats were used as the nondiabetic control model.

We performed an oral glucose tolerance test (OGTT) every 4 weeks. Before the test, the rats fasted for 16 h overnight, and 30% glucose solution (2 g/kg) was administered by gastric gavage. We collected the blood samples from the tail vein before glucose loading and 30, 60, and 120 min after glucose loading. Blood glucose levels were determined using an automatic blood glucose analyser. Plasma insulin concentrations were detected using radioimmunoassay. OLETF rats were classified into four stages according to the OGTT results [14] and 24 h urinary microalbumin (24 h UMA) levels: the normal glucose tolerance (NGT) stage, corresponding to a normal glucose level; the IGT stage, corresponding to a peak level of blood glucose > 16.8 mmol/L, or a blood glucose level > 11.1 mmol/L at 120 min; the DM stage, when both the above criteria were fulfilled; and the diabetic kidney disease (DKD) stage when the amount of 24 h UMA in OLETF rats was significantly greater than that in LETO rats. Tumour necrosis factor-*α* (TNF-*α*) and interleukin-6 (IL-6) serum levels were tested using enzyme-linked immunosorbent assay (ELISA). Eight OLETF rats were randomly selected and euthanized in their NGT, IGT, DM, and DKD stages, as the NGT, IGT, DM, and DKD groups, and the same-aged LETO rats were euthanized in the control group.

### 2.2. Retina Structure

1. Immunohistochemistry (IHC). Retina tissues were immediately fixed in 4% formalin and subsequently embedded in paraffin. The expression levels of HIF-1*α* (209601-1-AP, Proteintech), iNOS (18985-1-AP, Proteintech), NF-*κ*B (ab7970, Abcam), VEGF (19003-1-AP, Proteintech), ICAM-1 (ab124760, Abcam), and Occludin (ab31721, Abcam) were tested in the retina using IHC
2. Light microscopy. Retina tissues were fixed in 4% paraformaldehyde and embedded in paraffin. Tissue slices were cut at 4 *μ*m thickness, dewaxed in xylene, rehydrated in decreasing concentrations of ethanol in water, stained by haematoxylin and eosin (H&E), and examined using a light microscope
3. Transmission electron microscopy. Retina tissues were immediately placed in a fixative (2.5% glutaraldehyde and 1% osmium tetraoxide), dehydrated using graded alcohol and epoxypropane, and embedded in Epon 812. Ultrathin sections were cut using an ELICA ULTRACUT-R ultramicrotome and stained with uranyl acetate and lead citrate. The sections were examined using a HITACHI-7500 transmission electron microscope

### 2.3. Statistical Analysis

We performed the statistical analysis using the IBM SPSS Statistics 26.0 software. All normally distributed data were expressed as the mean ± standard deviation (SD), and all other data were expressed as the median (range). Differences among the three groups were analysed using one-way analysis of variance for normally distributed continuous parameters. If differences were observed, the LSD *t*-test was used for the comparison between the two groups. Nonnormally distributed data were compared using the Kruskal–Wallis test. If differences were observed, the Mann–Whitney test was used for the comparison between the two groups. A chi-square test was used to compare the differences in the measurement data. Results were considered statistically significant if the two-tailed *P* value was <0.05.

## 3. Results

### 3.1. The Biochemical Data in Different Stage of OLETF Rats

The fasting insulin (FINS) level and weight in the control and NGT group were not statistically different. However, the levels of the above two indicators were higher in the IGT stage than in the control group. Their levels fell in the DM and DKD groups but were still higher than those in the control group (*P* < 0.05).

The expression levels of TNF-*α* and IL-6 were increased in the IGT stage and continued to increase with the progression of the disease (*P* < 0.05). The levels of 24 h UMA were similar in the NGT, IGT, and DM groups but were higher in the DKD group than in the other groups (Table 1).

### 3.2. Significant Retinal Neuropathy Occurs in the IGT Stage

**Figure 1:**
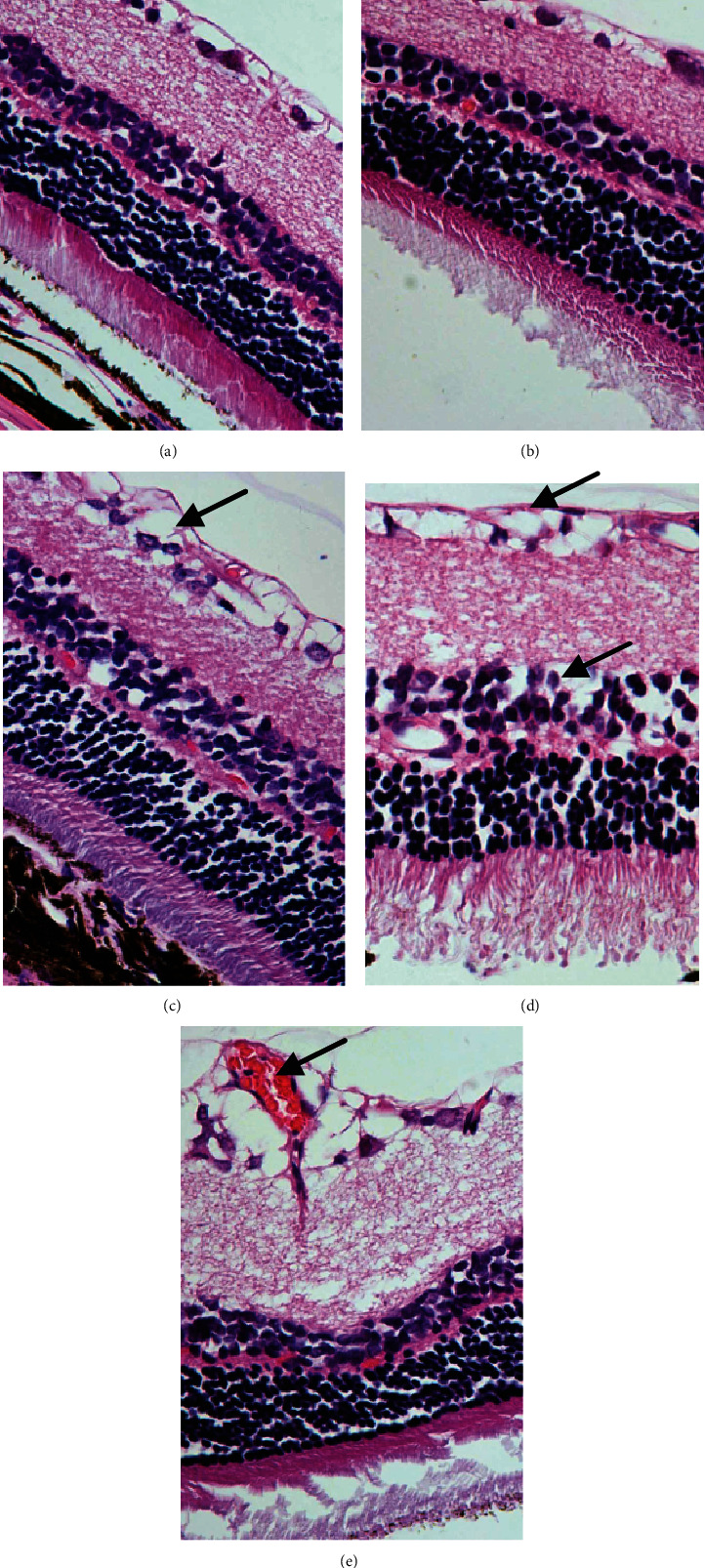
Pathological changes of the retina using H&E (×400). (a) The control group; the inner limiting membrane is clear, and the nerve fibre layer is sparse and regularly arranged; the ganglion cells are neatly arranged in a single layer, and the nuclei are large and round; the inner plexus layer is thicker and has a relatively distinct network structure; the inner granular layer is composed of 3-5 layers of cells; the nuclei are larger and appear slightly darker; the outer plexus layer is distinctly thinner than the inner plexus layer; the outer granular layer is thicker, consisting of 8-10 layers of tightly arranged cells with small, deeply stained nuclei. (b) The NGT group. (c) The IGT group. (d) The DM group. (e) The DKD group.

In the control group (Figure 1(a)) and NGT stage (Figure 1(b)), the structures of all the layers in the retina were normal. While in the IGT stage, significant damage had occurred to the retina, including interstitial oedema of the ganglion cell layer, the irregular surface of the inner limiting membrane, and the decreasing of retinal ganglion cells (RGCs) (Figure 1(c)). And in the DM stage (Figure 1(d)), all layers of the retina were structurally disordered, and intercellular substance oedema was conspicuous (as shown by the arrow). In the DKD stage (Figure 1(e)), the retinal ganglion cells were severely degenerated, and a new blood vessel was observed (as shown by the arrow).

**Figure 2:**
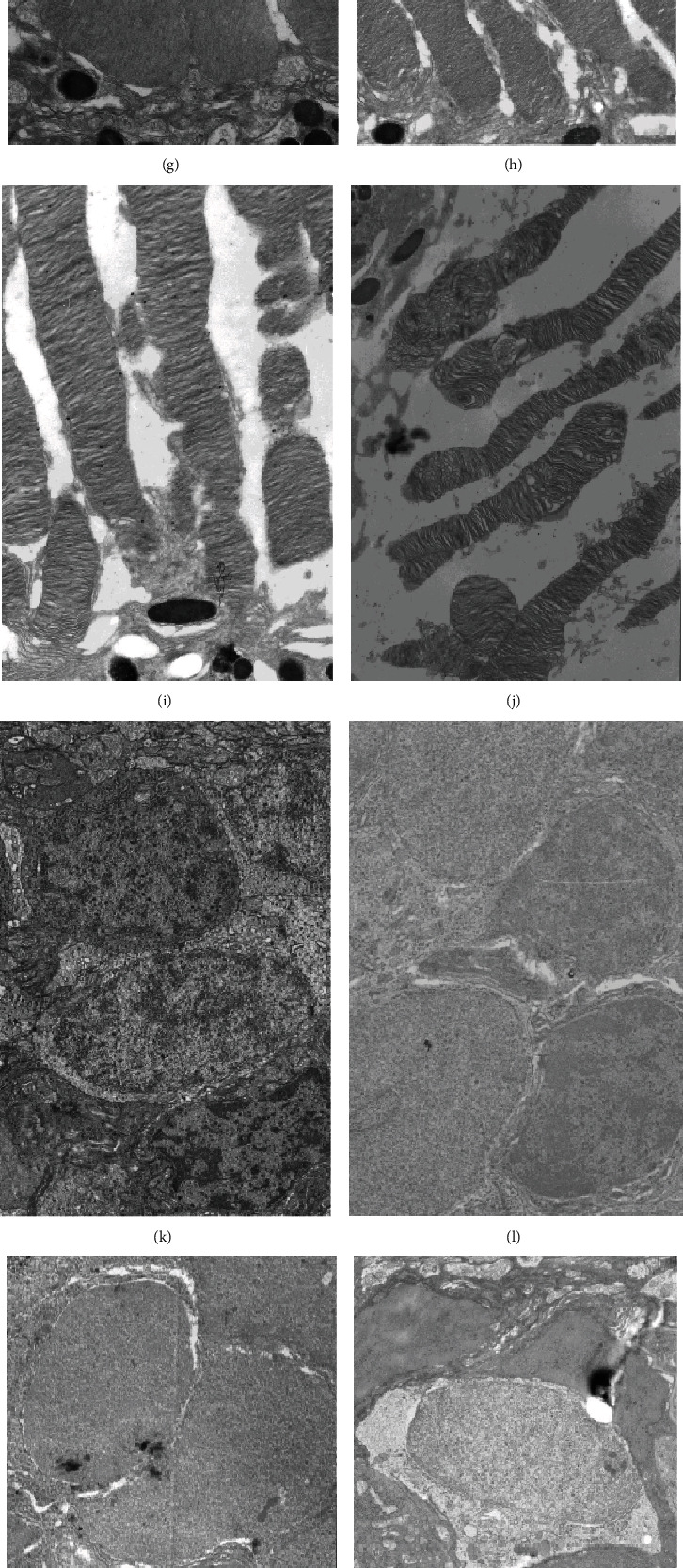
Pathological changes of the retina using transmission electron microscope. (a–e) The ganglion cell (×5000) of control, NGT, IGT, DM, and DKD stage, respectively; (f–j) the retinal disc (×10000) of control, NGT, IGT, DM, and DKD stage, respectively; (k–o) inner granular layer of the retina (×5000) in control, NGT, IGT, DM, and DKD stage, respectively; (p–t) outer granular layer of the retina (×5000) in control, NGT, IGT, DM, and DKD stage, respectively.

We then observed the ultrastructure of the retina using a transmission electron microscope. Compared with those in the control group, the rats in the NGT group had normal retinal morphology as well as ultrastructure (Figures 2(a), 2(b), 2(f), 2(g), 2(k), 2(l), 2(p), and 2(q)). However, abnormal morphological appeared in the ganglion cells at the IGT stage, including mitochondrial swelling, fuzzy crest, and vacuolization (Figure 2(c)). The cell membrane discs of visible light receptors were blurred and broken (Figure 2(h)), and oedema in the inner granular cells was observed (Figure 2(m)). The outer granular layer cells were irregularly arranged (Figure 2(r)). The above morphological changes gradually aggravated as the disease progressed (Figures 2(d), 2(e), 2(i), 2(j), 2(n), 2(o), 2(s), and 2(t)).

### 3.3. Immunohistochemical Staining Alteration of Inflammatory Markers, Cytokines, and HIF-1*α* in Different Stages

HIF-1*α*, NF-*κ*B, VEGF, and ICAM-1 were significantly upregulated in the IGT stage, while occludin was downregulated.

#### 3.3.1. Expression of HIF-1*α* and iNOS in the IGT Stage of OLETF Rats

**Figure 3:**
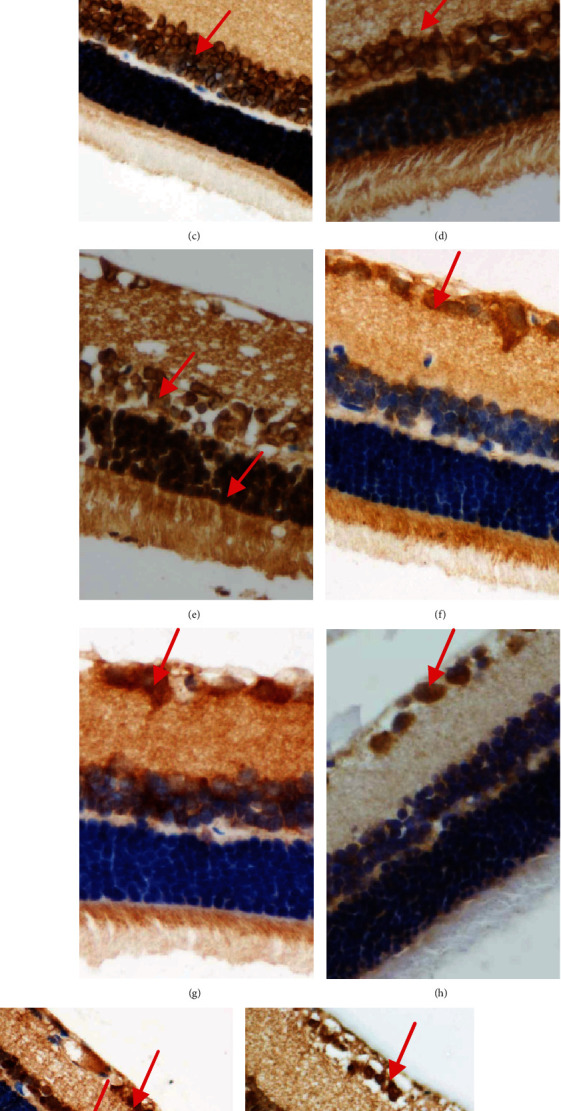
The immunohistochemical staining of HIF-1*α* and iNOS in retina of OLETF rats at different stages. (a–e) The expression of HIF-1*α* (×400) in the retina of control, NGT, IGT, DM, and DKD stage, respectively; (f–j) the expression of iNOS (×400) in the retina of control, NGT, IGT, DM, and DKD stage, respectively; (k, l) the quantification of HIF-1*α* and iNOS by Image J. ^a^*P* < 0.05 vs. the control group. ^b^*P* < 0.05 vs. NGT. ^c^*P* < 0.05 vs. IGT. ^d^*P* < 0.05 vs. DM.

Same as the control group (Figure 3(a)), HIF-1*α* was mainly expressed in retinal ganglion cell layer and low expressed in inner granular layer in the NGT group (Figure 3(b)). While in the IGT stage, the expression of HIF-1*α* increased significantly in inner granular layer (Figure 3(c)), and it further increased in the DM stage (Figure 3(d)). In the DKD stage, HIF-1*α* was expressed almost in the entire retina, including the outer granular layer (Figure 3(e)). Though the expression of iNOS has no significant difference between IGT stage (Figure 3(h)), NGT stage (Figure 3(g)), and control group (Figure 3(f)), it significantly increased in the DM stage (Figure 3(i)), and in the DKD stage, it was expressed almost in the entire retina (Figure 3(j)). The quantification of HIF-1*α* and iNOS is shown in Figures 3(k) and 3(l).

#### 3.3.2. Expression of NF-*κ*B, VEGF, and ICAM-1 in the IGT Stage of Rats

**Figure 4:**
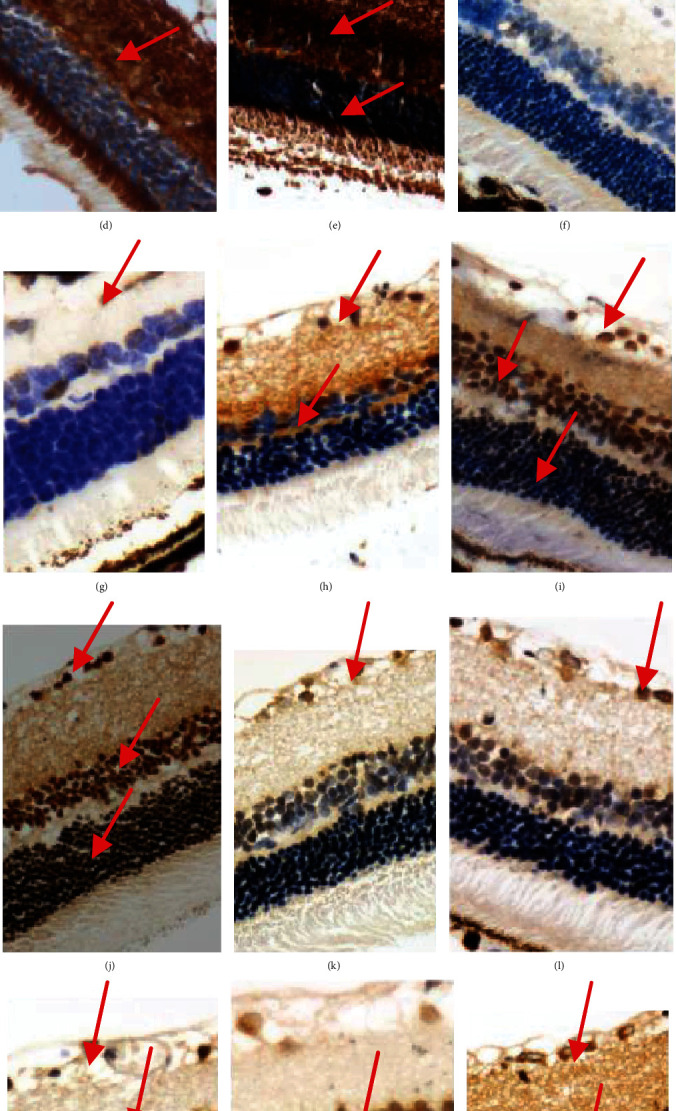
The immunohistochemical staining of NF-*κ*B, VEGF, and ICAM-1 in retina of OLETF rats at different stages. (a–e) The expression of NF-*κ*B (×400) in the retina of control, NGT, IGT, DM, and DKD stage, respectively, using IHC; (f–j) the expression of VEGF (×400) in the retina of control, NGT, IGT, DM, and DKD stage, respectively, using IHC; (k–o) the expression of ICAM-1 (×400) in the retina of control, NGT, IGT, DM, and DKD stage, respectively, using IHC; (p–r) the quantification of NF-*κ*B, VEGF, and ICAM-1. ^a^*P* < 0.05 vs. the control group. ^b^*P* < 0.05 vs. NGT. ^c^*P* < 0.05 vs. IGT. ^d^*P* < 0.05 vs. DM.

NF-*κ*B, VEGF, and ICAM-1 were low expressed in the retinal ganglion cell layer and the inner granular layer in the rats of the control group (Figures 4(a), 4(f), and 4(k)) and NGT stage (Figures 4(b), 4(g), and 4(l)). These inflammatory markers increased markedly in the IGT stage (Figures 4(c), 4(h), and 4(m)). And they further increased in the DM stage (Figures 4(d), 4(i), and 4(n)), and in the DKD stage, they were expressed almost in all the layers of the retina (Figures 4(e), 4(j), and 4(o)).The quantification of NF-*κ*B, VEGF, and ICAM-1 is shown in Figures 4(p)–4(r).

#### 3.3.3. Occludin Expression in the IGT Stage in Rats

**Figure 5:**
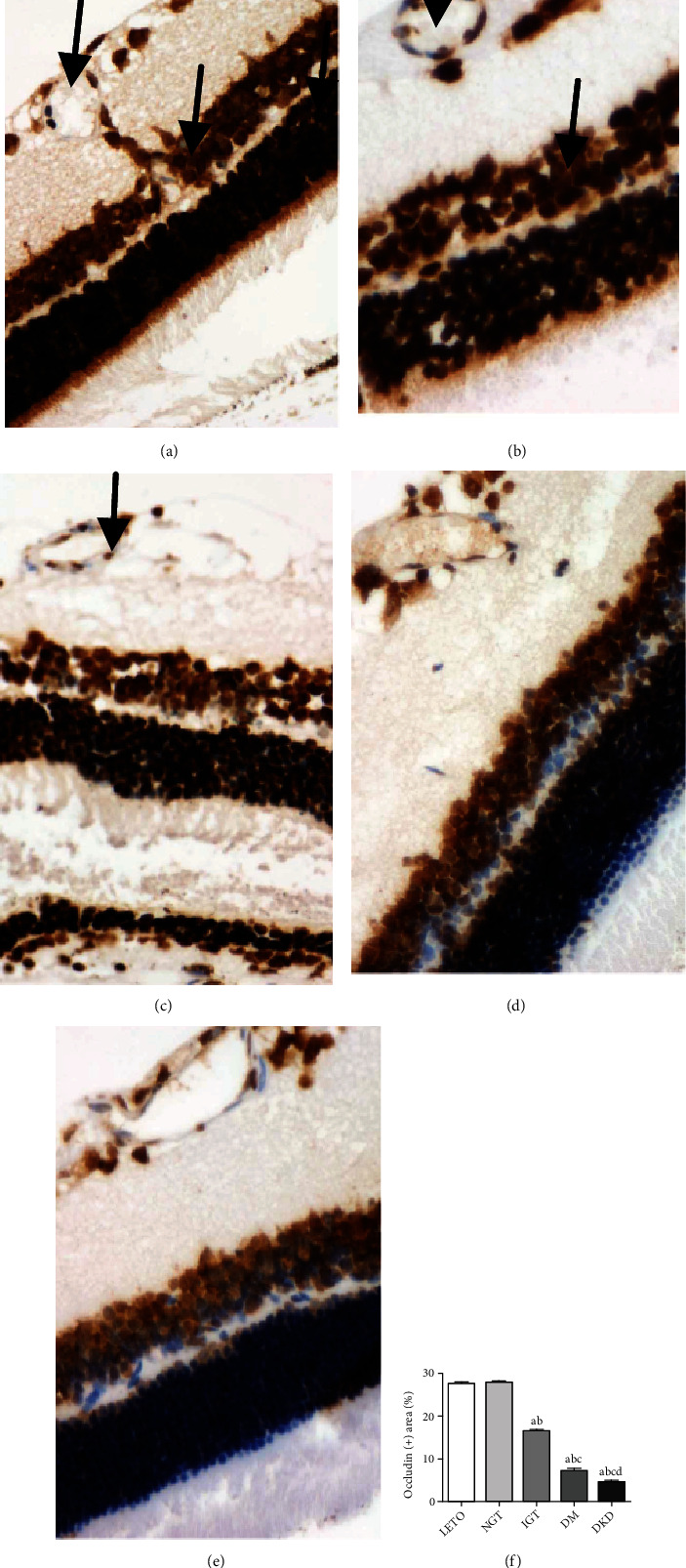
The immunohistochemical staining of occludin in retina of OLETF rats at different stages. (a–e) The expression of occludin (×400) in the retina of control, NGT, IGT, DM, and DKD stage, respectively, using IHC; (f) the quantification of occludin. ^a^*P* < 0.05 vs. the control group. ^b^*P* < 0.05 vs. NGT. ^c^*P* < 0.05 vs. IGT. ^d^*P* < 0.05 vs. DM.

Consistent with the control group (Figure 5(a)), the occludin was mainly expressed in the retinal ganglion cell layer and the inner granular layer in the NGT stage (Figure 5(b)). In the IGT stage, the expression of occludin declined in the ganglion cell layer (Figure 5(c)) and further decreased as the disease progressed (Figures 5(d) and 5(e)). The quantification of occludin is shown in Figure 5(f).

#### 3.3.4. The mRNA Levels of Inflammatory Markers, Cytokines, and HIF-1*α* in Retina of OLETF Rats at Different Stages

**Figure 6:**
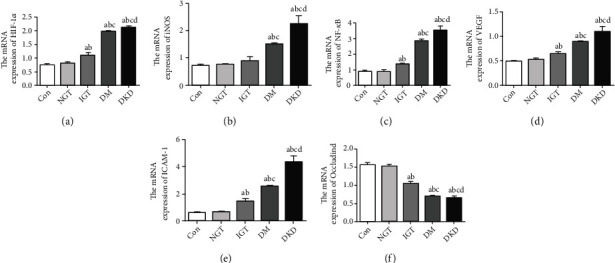
The mRNA levels of inflammatory markers, cytokines, and HIF-1*α* in retina of OLETF rats at different stages. ^a^*P* < 0.05 vs. the control group. ^b^*P* < 0.05 vs. NGT. ^c^*P* < 0.05 vs. IGT. ^d^*P* < 0.05 vs. DM.

Compared with the control group, in the IGT stage, the mRNA expression of HIF-1*α* (Figure 6(a)), iNOS (Figure 6(b)), NF-*κ*B (Figure 6(c)), VEGF (Figure 6(d)), and ICAM-1 (Figure 6(e)) was upregulated by 33.3%, 18.2%, 35.8%, 23.4%, 58.2%, respectively, while that of occludin (Figure 6(f)) was decreased by 32.7%.

## 4. Discussion

Our study observed the pathological changes occurring in the retina in the IGT stage, focusing on the damage to the ganglion and photoreceptor cells. In the retina, photoreceptors transmit visual information to bipolar cells, which process and pass it to RGCs. Next, the RGC axons travel through the optic nerve, telling the brain about the visual world [15]. The integrity of RGCs promises the normal function of the retina; however, abnormal environment such as hyperglycemia alters the structure or function of RGCs, and this kind of RGCs impairment is progressive in the development of subsequent DR [16].

Diabetic retinopathy is a neurovascular disease, one of the leading causes of severe vision loss. RGCs are damaged in diabetic retinopathy, producing cell function impairment and their subsequent loss [17].

DR was previously considered a microvascular complication of diabetes, but now, it is widely considered a neurovascular disease caused by neurovascular unit (NVU) injury. The recent view is that retinal neurodegeneration occurs before microvascular impairment especially the vulnerable neurons like RGCs; the destruction of the neural unit leads to the vascular unit injury and accelerates microvascular damage [18]. Studies have shown that diabetic retinal neuropathy is caused mainly by retinal photoreceptor lesions [19, 20]. In our study, we observed abnormal structures, including mitochondrial swelling, fuzzy crest, vacuolization in the ganglion cells, and blurred and broken retinal photoreceptor cells in the IGT stage. Interstitial oedema and degeneration of the ganglion cells in the retinal ganglion cell layer were also observed in the IGT stage. Neovascularity was not observed before the DKD stage. These results suggest that neurodegenerative changes may occur in the early stages of diabetes.

The blood retina barrier (BRB) is a structure that protects retinal cells and optic nerves from damage from cytotoxic substances and provides a stable metabolic environment for them. Structural damage and increased permeability of the BRB are involved in the pathogenesis of DR. However, the pathogenesis of DR is still unclear, as there is no single mechanism that can explain the destruction of BRB. A series of metabolic abnormalities, such as ischemia, hypoxia, inflammation, oxidative stress, and some cytokines, are thought to be involved in the BRB damage.

Recently, many studies have shown that the local, chronic low-grade inflammation is closely related to the onset and development of DR [21–23]. The activation and release of NF-*κ*B and other inflammatory cytokines play an important role in DR. It has been demonstrated that the expression of inflammatory markers such as IL-6, iNOS, and TNF-*α* is upregulated in the retina in DR [24–27].

Similarly, in our study, the expression of TNF-*α* and IL-6 was significantly increased in the IGT stage and continued to increase as the disease progressed. Normally, the levels of VEGF are low in the retina to maintain the integrity of retinal blood vessels. Abnormally, high levels of VEGF can stimulate endothelial cell migration and vascular proliferation, causing retinal oedema and exudation and leading to haemangioma. In our study, the expression of VEGF, ICAM-1, and NF-*κ*B was significantly upregulated in the IGT stage and continued to increase as the disease progressed. VEGF is one of the important cytokines that boosts the onset and development of DR. Of the many factors capable of upregulating VEGF, hypoxia is the most direct acting factor. Under hypoxic conditions, the expression of HIF-1*α* was upregulated, thus promoting the expression of VEGF. We also observed that the expression of HIF-1*α* and iNOS in the retina increased gradually, consistent with previous studies [28, 29]. As the disease progresses, the expression of VEGF, ICAM-1, and TNF-*α* gradually increases in the retina, which in turn downregulates the expression of occludin. As occludin is involved in the formation of tight junctions, it is closely related to the BRB. Reportedly, the expression of occludin can be significantly decreased in the DM rat model and in retinal vascular endothelial cells stimulated by high glucose [30], damaging the BRB and causing vascular leakage. We observed a decreased occludin expression in the retina in the IGT stage, which further decreased as the disease progressed, suggesting damage to the BRB. The above results suggest that inflammation and hypoxia may together participate in BRB damage, leading to DR. However, its exact pathogenesis needs to be investigated further.

In conclusion, the retinal neurodegeneration begins at an early stage. Therefore, early prevention and intervention are important for the treatment of diabetic retinopathy.

## Figures and Tables

**Table 1:** Biochemical data in the different stages of rats.

Group	Weight (g)	FBG (mmol/L)	FINS (uIU/L)	24 h UMA (*μ*g/24 h)	TNF-*α* (ng/L)	IL-6 (pg/mL)
Control	291.63 ± 4.93	5.74 ± 0.21	23.00 ± 1.60	151.68 ± 24.22	61.80 ± 12.92	43.13 ± 11.27
NGT	301.00 ± 16.95	5.59 ± 0.30	24.04 ± 2.52	145.73 ± 21.48	65.40 ± 10.71	41.76 ± 9.83
IGT	608.38 ± 34.12^ab^	5.86 ± 0.33	64.66 ± 2.85^ab^	163.43 ± 21.78	84.88 ± 20.62^ab^	115.06 ± 21.69^ab^
DM	586.50 ± 50.60^ab^	6.61 ± 1.05^abc^	33.53 ± 4.71^abc^	168.07 ± 21.95	103.66 ± 23.50^abc^	140.05 ± 32.89^abc^
DKD	563.14 ± 88.92^ab^	6.83 ± 0.41^abc^	29.35 ± 3.13^abcd^	224.21 ± 57.87^abcd^	106.42 ± 25.17^abc^	176.49 ± 32.30^abcd^

^a^ *P* < 0.05 vs. the control group. ^b^*P* < 0.05 vs. NGT. ^c^*P* < 0.05 vs. IGT. ^d^*P* < 0.05 vs. DM.

## Data Availability

The data used to support the findings of this study are available from the corresponding author upon request.
